# Update on the enigmatic *Pteroceraphron mirabilipennis* Dessart, 1981 (Hymenoptera, Ceraphronidae): description of male and first record from the Neotropics, including first DNA barcodes

**DOI:** 10.3897/BDJ.14.e189669

**Published:** 2026-04-07

**Authors:** Tobias Salden, Jonah A. Schneider, István Mikó, Ralph S. Peters

**Affiliations:** 1 Leibniz Institute for the Analysis of Biodiversity Change, Museum Koenig, Bonn, Germany Leibniz Institute for the Analysis of Biodiversity Change, Museum Koenig Bonn Germany; 2 University of New Hampshire, Durham, United States of America University of New Hampshire Durham United States of America

**Keywords:** Ceraphronoidea, Costa Rica, DNA barcode, male genitalia

## Abstract

**Background:**

*Pteroceraphron* is a morphologically distinct genus of ceraphronid wasps known only from few female specimens of two species, *Pteroceraphron mirabilipennis* Dessart, 1981 from the Nearctic Region and *P. apoorva* Bijoy & Rajmohana, 2021 from the Oriental Region.

**New information:**

Our study provides the first report and description of male specimens of the genus *Pteroceraphron*, the first record of *Pteroceraphron mirabilipennis* Dessart, 1981 from the Neotropics (Costa Rica), including the first DNA barcode data, as well as an updated diagnosis for the genus.

## Introduction

Ceraphronidae is an often collected and severely understudied family of parasitoid wasps comprising over 410 described species (Johnson and Musetti 2004, Silva and Feitosa 2017, Caterino and Recuero 2023, Haas-Renninger et al. 2023, Salden and Peters 2023, Verheyde et al. 2023, Ghafouri Moghaddam et al. 2025).

*Pteroceraphron* Dessart, 1981 is a genus of Ceraphronidae, known so far from only few female specimens in two species, *P. mirabilipennis* Dessart, 1981, described from Canada (Dessart 1981), but also recorded from various locations in the eastern USA (Mikó et al. 2016) and the recently described *P. apoorva* Bijoy & Rajmohana, 2021 from India (Bijoy and Rajmohana 2021). Species of *Pteroceraphron* are small-sized (approx. 1 mm) and both possess lanceolate fore wings equipped with elongate marginal setae that make them easily distinguishable from other genera of Ceraphronidae. The biology as well as males and the species’ distribution ranges are currently unknown.

Here, we provide an update on our knowledge of the genus and, specifically, *P. mirabilipennis*, based on material examined from Costa Rica that includes the first known males of the genus, as well as the first nucleotide sequence data (DNA barcodes). In addition, when considering both described species, a refined genus diagnosis is necessary that we provide herein.

## Materials and methods

**Handling of specimens and imaging**

Six specimens deposited at the Centre for Biodiversity Genomics (CBG) were examined, which form the BIN BOLD:ADB3043. Three additional specimens of the same BIN with the following process IDs were not available, i.e. they were not examined morphologically and were not used for the barcode consensus sequence (BOLD Process IDs CRLDC12609-24 (male), CRLDC3207-24 (female), CRLDC41515-24 (male)). All examined specimens were collected in Costa Rica with Malaise traps and preserved in ethanol (Janzen and Hallwachs 2019).

The male specimen CBG-A59635-C04 was card mounted. The male specimen ZFMK-HYM-00042676 and the female specimen ZFMK-HYM-00042675 were transferred into glycerol on concave microscope slides. Examinations and dissections were done under a Leica M205C stereomicroscope with a Leica Planapo 1.0 ×, M-series (WD 61.5 mm) objective and Leica PI 10 ×/23 oculars. Measurements were obtained using a calibrated Leica ocular scale. Male genitalia of ZFMK-HYM-00042676 were dissected using Dumont Style 5 Inox 02 forceps and a minutien pin (100 µm diameter). The Waterston’s evaporatorium of the male specimen ZFMK-HYM-00042676 and the female specimen ZFMK-HYM-00042675 were dissected using two minuten pins (100 µm diameter). The 6^th^ metasomal tergites with the Waterston’s evaporatorium were separated from the rest of the body and transferred into small glycerol droplets on microscope slides. Cover slides were placed left and right of the droplet and a third one on top of these two. The lateral slides were subsequently removed one by one to allow the top one to smoothly cover the droplet.

The female holotype of *Pteroceraphron mirabilipennis*, deposited in the Canadian National Collection of Insects, Arachnids and Nematodes (CNC), Ottawa, was imaged with an Olympus CX41 microscope with a Canon EOS 70D camera attached. All images were then aligned and stacked using Zerene Stacker Version 1.04 Build T201706041920 (Zerene Systems LLC, Richland, WA, USA) and morphologically examined. Further images were taken using a Keyence VHX-7100 microscope.

Terminology for body morphology, including male genitalia, female terminalia and Waterson’s evaporatoria, is based on Mikó and Deans (2009), the Hymenoptera Anatomy Ontology (HAO) (Yoder et al. 2010), as well as on Mikó et al. (2013), Ernst et al. (2013), Ulmer et al. (2021) and Salden and Peters (2023). Additionally, two new characters of the Waterston's evaporatorium (WE) are defined herein: WE length, defined as the median anatomical line between the anteriormost point of the acrotergal calyx and the posteriormost point of the evaporatorium and WE length/median T6 length, defined as the WE length divided by the length of the metasomal tergite 6, measured as the median anatomical line between the anteriormost point of the acrotergal calyx and the posterior margin of the tergite.

**DNA sequencing and consensus sequence**

DNA barcode sequences were generated at the Canadian Centre for DNA Barcoding (CCDB). The DNA extraction followed the protocol by Ivanova et al. (2006). Amplification of the DNA barcode region was performed according to the CCDB CO1 Amplification Protocol using the primers LepF1 and LepR1 (Hebert et al. 2004). Sequencing followed the CCDB Sequencing Protocol and sequence clean-up was carried out following the protocol of Anonymous (2007). All CCDB protocols are available online at https://ccdb.ca/resources/ (accessed on 21 January 2026).

The sequences were aligned with MUSCLE (Edgar 2004) in AliView v.1.28 (Larsson 2014) and the consensus sequence was extracted in UGENE v.52.1 using the default strict consensus approach (Okonechnikov et al. 2012). This consensus sequence represents a summary of the available sequence information of the BIN BOLD:ADB3043 and is useful for the comparison of DNA barcodes. However, it does not capture the complete or true extent of intraspecific sequence variation. Based on the resulting alignment, we generated a distance matrix with MEGA version 12.0.10 (Kumar et al. 2024) to assess the maximum intraspecific distance (Suppl. material 1).

## Taxon treatments

### Pteroceraphron

Dessart, 1981

700F7BF4-7F12-5976-AFEF-6281F4EDDCF9

Pteroceraphron mirabilipennis Dessart, 1981

#### Diagnosis

**Figure 1.**
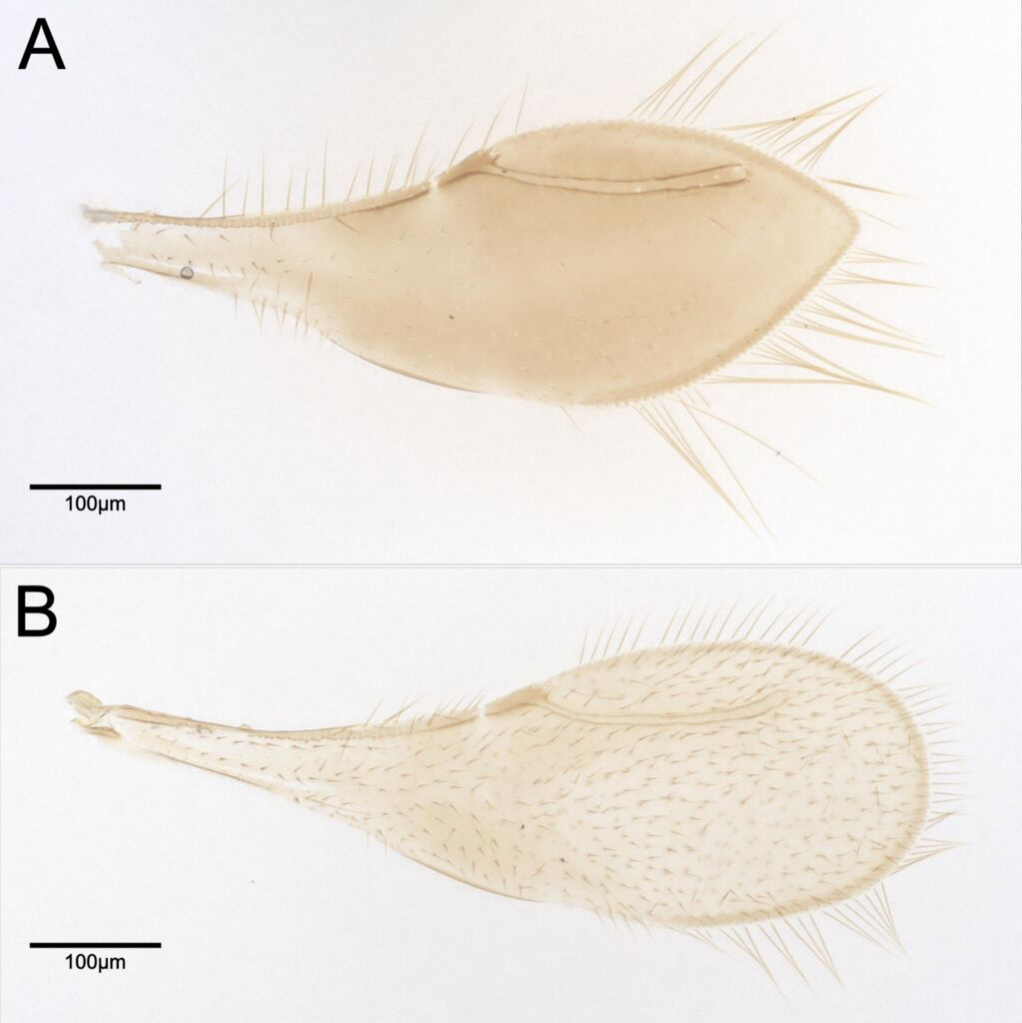
Right fore wing of *Pteroceraphron mirabilipennis*. **A** female (ZFMK-HYM-00042675); **B** male (ZFMK-HYM-00042676).

*Pteroceraphron* differs from all other ceraphronoid genera by the fore wing uniformly melanised and lanceolate in female specimens (Fig. 1A); in both sexes, the stigmal vein is elongate and the wing margin bears elongate marginal setae (Fig. 1).

#### Distribution

Nearctic: Canada, USA.

Neotropical: Costa Rica.

Oriental: India

#### Biology

Unknown.

### Pteroceraphron mirabilipennis

Dessart, 1981

1E2963F3-15C6-5408-AABB-0FFB52A06117

#### Materials

- **Type status:** Other material. **Occurrence:** catalogNumber: ZFMK-HYM-00042675; recordedBy: D. Janzen, W. Hallwachs; sex: female; occurrenceID: EC2786FB-E985-5695-8A99-0AFF3C08BAD4; **Taxon:** family: Ceraphronidae; genus: Pteroceraphron; specificEpithet: mirabilipennis; scientificNameAuthorship: Dessart 1981; **Location:** country: Costa Rica; stateProvince: Guanacaste; county: Liberia; locality: Sector Pailas, Pailas Dos; verbatimLatitude: 10.764; verbatimLongitude: -85.335; **Identification:** identifiedBy: Tobias Salden; dateIdentified: 2025; **Event:** samplingProtocol: Malaise Trap; eventDate: 14-08-2014; **Record Level:** institutionCode: ZFMK
- **Type status:** Other material. **Occurrence:** catalogNumber: CBG-A08578-F07; recordedBy: D. Janzen, W. Hallwachs; sex: female; occurrenceID: CD141FDF-89E7-5629-BD79-8F093D82C071; **Taxon:** family: Ceraphronidae; genus: Pteroceraphron; specificEpithet: mirabilipennis; scientificNameAuthorship: Dessart 1981; **Location:** country: Costa Rica; stateProvince: Alajuela; locality: Sector San Cristobal; verbatimLatitude: 10.88; verbatimLongitude: -85.389; **Identification:** identifiedBy: Tobias Salden; dateIdentified: 2025; **Event:** samplingProtocol: Malaise Trap; eventDate: 20-05-2019; **Record Level:** institutionCode: BIOUG
- **Type status:** Other material. **Occurrence:** catalogNumber: CBG-A33551-A08; recordedBy: D. Janzen, W. Hallwachs; sex: female; occurrenceID: 832B3E77-BF67-5A75-97E8-254DDF37920C; **Taxon:** family: Ceraphronidae; genus: Pteroceraphron; specificEpithet: mirabilipennis; scientificNameAuthorship: Dessart 1981; **Location:** country: Costa Rica; stateProvince: Alajuela; locality: Liceo Dos Rios; verbatimLatitude: 10.8930855; verbatimLongitude: -85.3794; **Identification:** identifiedBy: Tobias Salden; dateIdentified: 2025; **Event:** samplingProtocol: Malaise Trap; eventDate: 04-07-2023; **Record Level:** institutionCode: BIOUG
- **Type status:** Other material. **Occurrence:** catalogNumber: ZFMK-HYM-00042676; recordedBy: D. Janzen, W. Hallwachs; sex: male; occurrenceID: 8A204DFC-76E5-5432-B688-28A1F6463AAD; **Taxon:** family: Ceraphronidae; genus: Pteroceraphron; specificEpithet: mirabilipennis; scientificNameAuthorship: Dessart 1981; **Location:** country: Costa Rica; stateProvince: Alajuela; locality: Liceo Dos Rios; verbatimLatitude: 10.8930855; verbatimLongitude: -85.3794; **Identification:** identifiedBy: Tobias Salden; dateIdentified: 2025; **Event:** samplingProtocol: Malaise Trap; eventDate: 13-06-2023; **Record Level:** institutionCode: ZFMK
- **Type status:** Other material. **Occurrence:** catalogNumber: CBG-A40264-H04; recordedBy: W. Porras; sex: female; occurrenceID: 259ED8C1-EDB8-5FF6-A668-185C12FE8753; **Taxon:** family: Ceraphronidae; genus: Pteroceraphron; specificEpithet: mirabilipennis; scientificNameAuthorship: Dessart 1981; **Location:** country: Costa Rica; stateProvince: Alajuela; locality: Nectandra Cloud Forest Reserve; verbatimLatitude: 10.187778; verbatimLongitude: -84.50806; **Identification:** identifiedBy: Tobias Salden; dateIdentified: 2025; **Event:** samplingProtocol: Malaise Trap; eventDate: 07-07-2023; **Record Level:** institutionCode: BIOUG
- **Type status:** Other material. **Occurrence:** catalogNumber: CBG-A59635-C04; recordedBy: D. Janzen, W. Hallwachs; sex: male; occurrenceID: F4DB365B-9792-5A17-9DB3-552052BAA232; **Taxon:** family: Ceraphronidae; genus: Pteroceraphron; specificEpithet: mirabilipennis; scientificNameAuthorship: Dessart 1981; **Location:** country: Costa Rica; stateProvince: Guanacaste; county: La Cruz; locality: Sector Pitilla; verbatimLatitude: 10.98906; verbatimLongitude: -85.42414; **Identification:** identifiedBy: Tobias Salden; dateIdentified: 2025; **Event:** samplingProtocol: Malaise Trap; eventDate: 10-10-2023; **Record Level:** institutionCode: BIOUG
- **Type status:** Holotype. **Occurrence:** catalogNumber: CNC N°17175; recordedBy: E. Sigler; sex: female; occurrenceID: F932A576-E5F5-5169-AD87-6EC07999198A; **Taxon:** family: Ceraphronidae; genus: Pteroceraphron; specificEpithet: mirabilipennis; scientificNameAuthorship: Dessart 1981; **Location:** country: Canada; stateProvince: Ontario; locality: St. Lawrence national Park; **Identification:** identifiedBy: Paul Dessart; **Event:** samplingProtocol: pan trap; eventDate: 16-07-1975; **Record Level:** institutionCode: CNC

#### Description

**Male (n = 2). Body length**: 0.58–0.63 mm.

**Figure 2.**
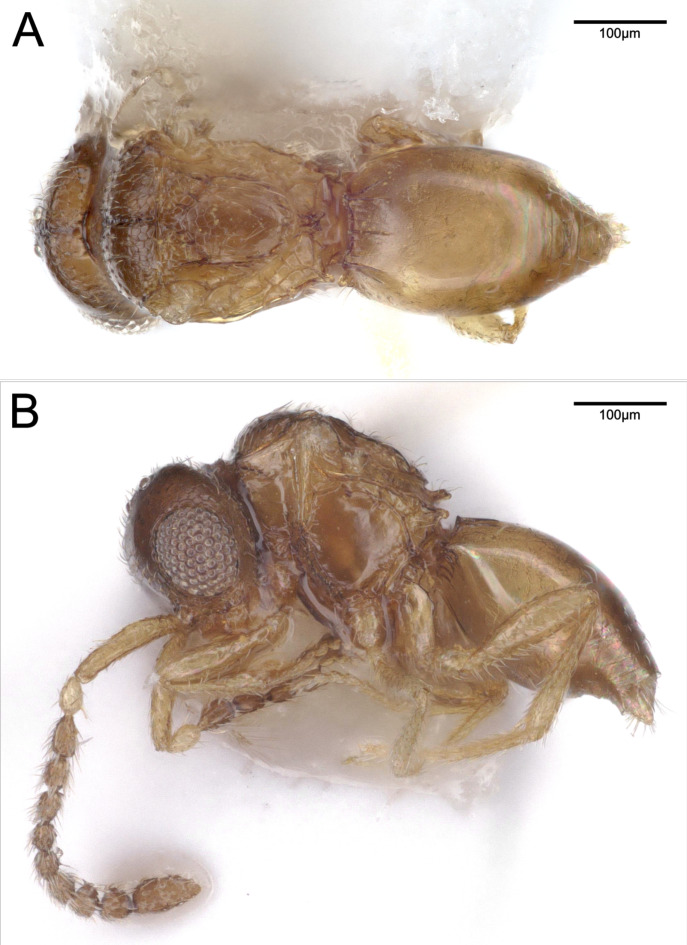
Male *Pteroceraphron mirabilipennis* (CBG-A59635-C04) habitus with removed wings. **A** dorsal view; **B** lateral view.

**Colour** (Fig. 2): Body yellowish, except head, flagellum, mesonotum and posterior third of metasoma light brown. Pedicel light yellow.

**Antenna, head** (Fig. 2): F2–F8 moniliform, scape length 3.1 × F1 length and 1.8 × F9 length. Preoccipital lunula present. Preoccipital furrow present. Preoccipital furrow ends anteriorly at POL. Preoccipital carina slightly indicated. Occipital carina present. Subocellar sulcus present.

**Mesosoma, fore wing, metasoma** (Fig. 2, Fig. 1B): Scutoscutellar sulcus and transscutal articulation not adjacent, interaxillar sulcus present, broad. Mesoscutellum distinctly rounded with U-shaped carina. Anteromedian projection of the metanoto-propodeo-metapecto-mesopectal complex short and slightly bifurcated. Dorsal region of anteromedian projection of the metanoto-propodeo-metapecto-mesopectal complex slightly setose. Mesometapleural sulcus absent. Wing length 0.70 mm. Wing not lanceolate and with elongate marginal setae on the wing margin present. Stigmal vein elongate and slightly curved. Stigmal vein length 4.3 × pterostigma marginal length. Transverse carina of syntergite distinct. Three distinct and six less distinct basal, longitudinal carinae on syntergum.

**Figure 3.**
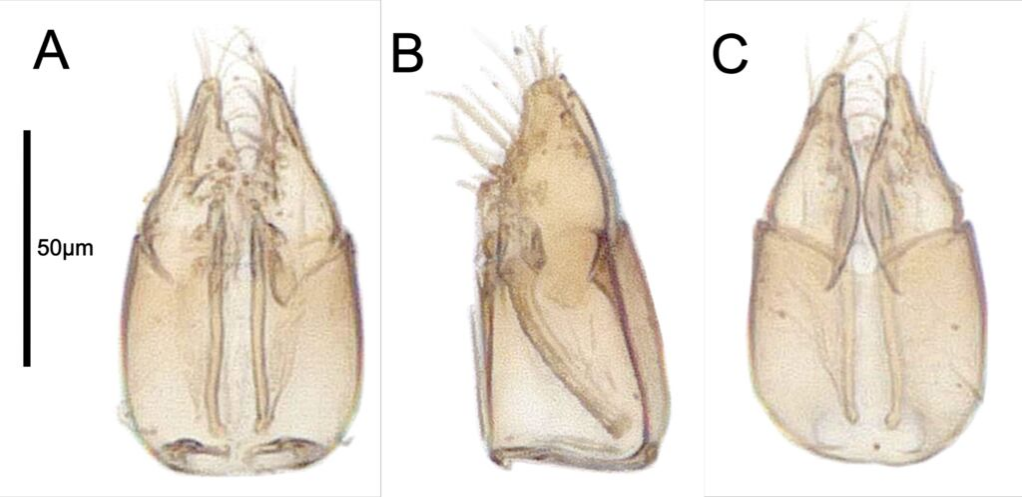
Male genitalia of *Pteroceraphron mirabilipennis* (ZFMK-HYM-00042676). **A** ventral view; **B** lateral view; **C** dorsal view.

**Male Genitalia (ZFMK-HYM-00042676)** (Fig. 3): Gvc length slightly less than one quarter of mesonotum length. Gvc width 1.02 × gvc length; distoventral margin of gvc descending proximomedially, distodorsal margin of gvc slightly descending proximomedially. Harpe/gvc index 0.74; harpe triangular; lateral margin of harpe straight and converging distomedially; dorsomedial margin of harpe convex in basal three-quarters and almost touching, straight in apical quarter and orientated distally; ventral margin of harpe straight in basal half and concave in apical half; dorsal margin of harpe slightly convex. Harpe with at least four median setae orientated distomedially and distoventrally, being slightly longer than half of harpe length; harpe with at least four apical setae orientated distally and slightly distoventrally; harpe with one to two lateral setae close to apex, orientated distoventrally and slightly distolaterally. Gonossiculus and aedeagus indistinct.

**Figure 4.**
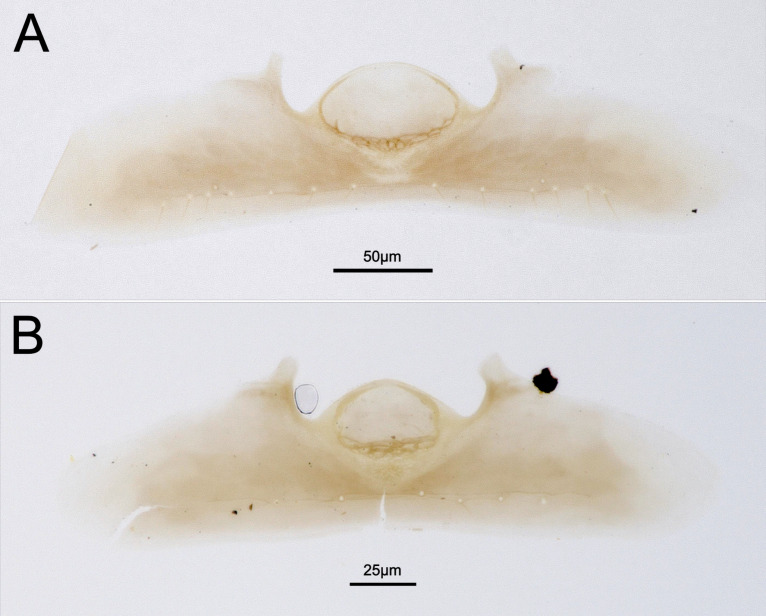
Waterston’s evaporatorium of *Pteroceraphron mirabilipennis*. **A** female (ZFMK-HYM-00042675); **B** male (ZFMK-HYM-00042676).

**Waterston’s evaporatorium (ZFMK-HYM-00042676)** (Fig. 4B): Acrotergal calyx distinctly convex. Evaporatorium with basomedial constriction, the basal cells covered by a proximomedian lamella and only faintly discernible under the microscope. Bulla convex anteriorly and straight posteriorly; laterally parallel. WE length/median T6 length 0.73 (including basomedial constriction to the WE length). Tergal apodemes broad and converging distally. Six setae in caudal setal row. Campaniform sensillae absent.

**Figure 5.**
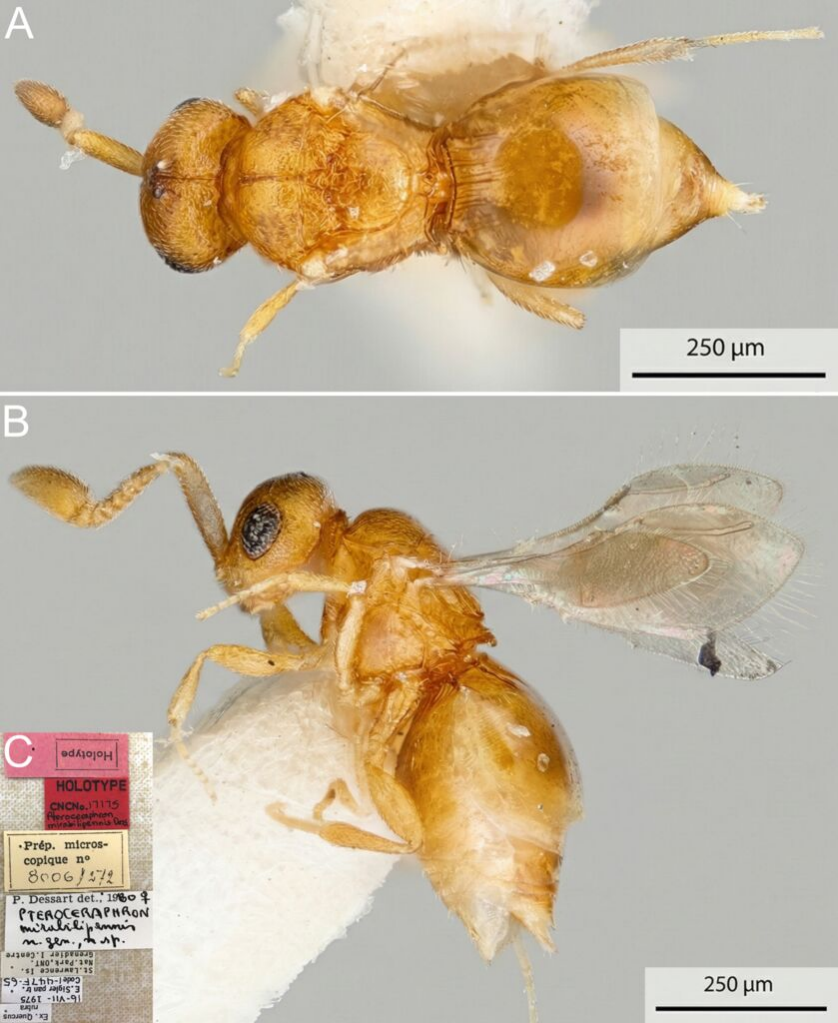
Female holotype of *Pteroceraphron mirabilipennis* (CNC N°17175). **A** dorsal view; **B** lateral view; **C** label data.

**Female.** Female specimens are larger than male specimens (Fig. 2, Fig. 5). The head height relative to the maximum eye diameter is distinctly smaller in male specimens, for example, CBG-A59635-C04 with 1.7, than in female specimens, for example, ZFMK-HYM-00042675 with 2.3 and the anteromedian projection of the metanoto-propodeo-metapecto-mesopectal complex is distinctly more bifurcated in female specimens (Fig. 2B, Fig. 5). The fore wing of female specimens is uniformly melanised and lanceolate and equipped with elongate marginal setae (Fig. 1). The antennae are sexually dimorphic, i.e. male flagellomeres are nearly uniformly wide with F2–F8 moniliform and female flagellomeres F1–F6 are nearly uniformly wide with F7 being widened and F8 being distinctly enlarged (Fig. 2B, Fig. 5). The Waterston’s evaporatorium of the female specimen (examined in specimen ZFMK-HYM-00042675) has a more oval bulla and the caudal setal row is equipped with 14 setae and one pair of campaniform sensillae is present (six setae and no campaniform sensillae in male) (Fig. 4). The WE length/median T6 length of the female is 0.83 (0.73 in male) and the basomedial cells are slightly less sclerotised in the female specimen (Fig. 4).

**Figure 6.**
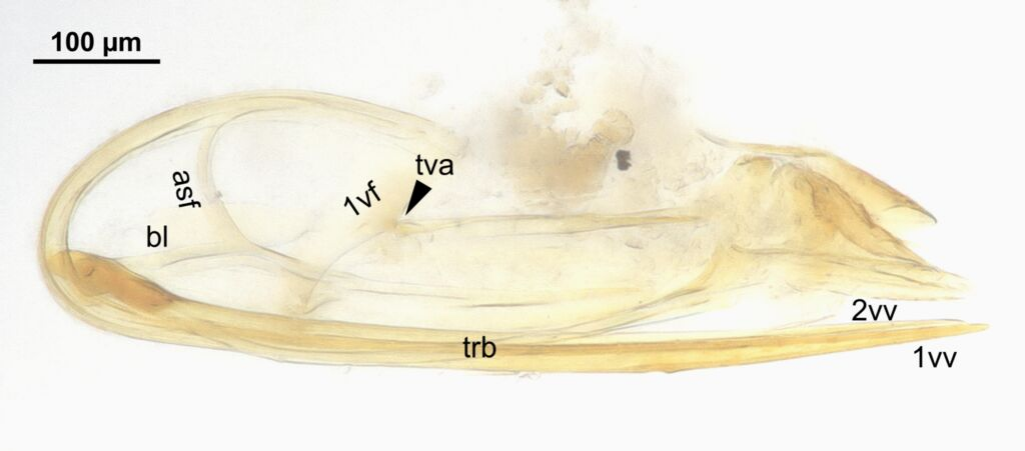
Female terminalia of *Pteroceraphron mirabilipennis* (ZFMK-HYM-00042675) with anterior section of dorsal flange of the second valvifer (asf), basal line of the second valvifer (bl), tergo-valvifer articulation (tva), terebra (trb), first valvifer (1vf), first valvulae (1vv) and second valvulae (2vv), lateral view.

The female terminalia is equipped with the tergo-valviferal articulation (tva) on the posterior margin of the first valvifer (1vf) located in the middle (Fig. 6). The first valvula (1vv) is gradually tapering distally and the second valvula (2vv) is almost as long as the first valvula. The basal line of the the second valvifer (bl) and the anterior section of dorsal flange of the second valvifer (asf) are distinct. The terebra (trb) is as long as the ovipositor assembly (Fig. 6).

#### Distribution

Nearctic: Canada, USA.

Neotropical: Costa Rica.

#### Biology

Unknown

#### DNA barcode

Maximum intraspecific distance: 1.02% (n = 6).

Consensus sequence: 661 bp

5’-ATTACTGTATTTTATTTTTGCTTTATGAGCAGGAATGGTAGGAGCAAGACTTAGGCTATTAATTCGTCTTGAACTAAGAAGTCCCCCTAATAACCTCATTAACAACGATCAAATTTATAATTCCATTATTACAGCCCATGCTTTTATTATAATTTTTTTTTTAGTTATACCAATTATATTAGGAGGGTTTGGAAACTGACTACTCCCTTTAATAATTGGAGGACCCGATATAGCTTTCCCTCGAATAAATAATATAAGATTTTGGTTACTCCCCCCTTCATTATTTATCTTAATTAATGGAATAATCTCTGACTCAGGAACTGGAGCAGGATGRACTTTATACCCTCCTTTRACATCAAATATTAATCATAATGGGATTTCAATAGACTTAACAATTTTCTCTTTACATATTGCAGGGATTAGATCAATTATAGGATCAATCAATTTTTTAGTAACCTTATATATAATAAATCCAAAATCAACAAAACATGAAATACTCCCATTATTCTGCTGATCAGTAGCTATCACAACAATTCTTTTAATTTTATCTTTACCTGTATTAGCAGGAGCCATTACAATAATCCTTACAGATCGAAATTTAAATACATCATTTTTTGATCCCAGAGGAGGAGGAGACCCTGTACTTTATCAGCATTTATTT -3’.

## Discussion

*Pteroceraphron* is a morphologically distinctive genus within Ceraphronidae, solely distinguished by the lanceolate fore wings of the females. All additional characters that were previously considered diagnostic, such as an enlarged last flagellomere, bifurcated anteromedian projection of the metanoto-propodeo-metapecto-mesopectal complex and three longitudinal carinae on the first metasomal tergum (Mikó et al. 2016), can no longer reliably define the genus, following the description of the Oriental species *P. apoorva* Bijoy & Rajmohana, 2021, which lacks these characters (Bijoy and Rajmohana 2021). Bijoy and Rajmohana (2021) also give a diagnosis under the genus name, but list in this diagnosis also antennal characters specific to *P. apoorva*, i.e. it is not clear of what they provide is a diagnosis of the species or of the genus. Thus, this diagnosis does not provide sufficient clarity. In addition, we here describe males of *Pteroceraphron* for the first time, clarifying female-specific characters. Both aspects led us to formally update the genus diagnosis.

To date, only a few records of *Pteroceraphron* have been published, which may reflect either true rarity or simply the absence of targeted collecting efforts for this lineage (Bijoy and Rajmohana 2021, Bennett et al. 2024). The newly-discovered male of *P. mirabilipennis* is not clearly morphologically distinguishable from species of the genus *Ceraphron*. Association with the corresponding females was done through the similarity of their DNA barcode sequences. It is possible that male *Pteroceraphron* have been collected and documented before, but were labelled as *Ceraphron*. Morphologically, both sexes of *P. mirabilipennis* can be associated by the structure of the Waterston’s evaporatorium (WE). Both sexes possess a distinct WE with a large bulla, distally converging, broad tergal apodemes and a basomedial constriction. The basomedial constriction shows basal cells that are smaller than the setal sockets, whereas the apical cells are larger, i.e. we found polymorphic evaporatorium cells, in contrast to Ulmer et al. (2021), who reported for this species uniformly-sized cells that are larger than the setal bases. In accordance with Ulmer et al. (2021), we observed stronger sclerotisation of the basomedial cells in male specimens compared to female specimens. In contrast to the female, we did not find campaniform sensillae in the examined male specimen. These characters might be subject to intraspecific variation (something we also see in other Ceraphronidae taxa (Schneider et al., unpublished data)). More specimens will have to be examined to validate sex-specific differences and specify the description of the WE. Additionally, the potential use of WE characters for the genus diagnosis will require both larger sample size and the inclusion of the second species, *P. apoorva*.

When re-checking BOLD, which served as a valuable resource for this study, we found five additional specimens of *Pteroceraphron* from Costa Rica, based on the basic specimen images available (Anonymous 2024). They form two different BINS which might indicate the presence of additional species (BOLD:AEN8173 (two males, one female); BOLD:AES5193 (one male, one female)). Specimens could not be examined in detail since we only found them when this study was almost completed.

Overall, the available data (Dessart 1981, Mikó et al. 2016; and results herein) suggest that *P. mirabilipennis* is widely distributed across the Nearctic and the northern Neotropics (Mesoamerican) Region. Nevertheless, we still lack detailed information on the species’ full distribution range, its biology, as well as the phylogenetic relationships of *Pteroceraphron* within Ceraphronidae. Continued searches and additional records from across the globe are needed to improve our understanding of these enigmatic ceraphronid wasps. This update including description of males, refined genus diagnosis and the first DNA barcodes with reliable names can help reach this goal.

## Supplementary Material

XML Treatment for Pteroceraphron

XML Treatment for Pteroceraphron mirabilipennis

E158E76C-548A-5664-AE35-90363774A961 10.3897/BDJ.14.e189669.suppl1 Supplementary material 1 distance_matrix_BIN_BOLD:ADB3043 Data type distance_matrix_CO1 Brief description distance matrix of six available CO1 sequences of the BIN BOLD:ADB3043. File: oo_1557887.xls https://binary.pensoft.net/file/1557887 Tobias Salden
